# Adsorption of Atomic Hydrogen on Hydrogen Boride Sheets Studied by Photoelectron Spectroscopy

**DOI:** 10.3390/ma17194806

**Published:** 2024-09-29

**Authors:** Heming Yin, Jingmin Tang, Kazuki Yamaguchi, Haruto Sakurai, Yuki Tsujikawa, Masafumi Horio, Takahiro Kondo, Iwao Matsuda

**Affiliations:** 1 The Institute for Solid State Physics, The University of Tokyo, Kashiwa 277-8581, Chiba, Japan; yin-heming200@g.ecc.u-tokyo.ac.jp (H.Y.);; 2 Department of Materials Science and Tsukuba Research Center for Energy Materials Science, Institute of Pure and Applied Sciences, University of Tsukuba, Tsukuba 305-8573, Ibaraki, Japan; takahiro@ims.tsukuba.ac.jp; 3 The Advanced Institute for Materials Research, Tohoku University, Sendai 980-8577, Miyagi, Japan

**Keywords:** 2D material, hydrogen boride sheets, atomic hydrogen adsorption, photoelectron spectroscopy

## Abstract

Hydrogen boride (HB) sheets are emerging as a promising two-dimensional (2D) boron material, with potential applications as unique electrodes, substrates, and hydrogen storage materials. The 2D layered structure of HB was successfully synthesized using an ion-exchange method. The chemical bonding and structure of the HB sheets were investigated using Fourier Transform Infrared (FT–IR) spectroscopy and Transmission Electron Microscopy (TEM), respectively. X-ray photoelectron spectroscopy (XPS) was employed to study the chemical states and transformation of the components before and after atomic hydrogen adsorption, thereby elucidating the atomic hydrogen adsorption process on HB sheets. Our results indicate that, upon atomic hydrogen adsorption onto the HB sheets, the B-H-B bonds were broken and converted into B-H bonds. This research highlights and demonstrates the changes in chemical states and component transformations of the boron element on the HB sheets’ surface before and after atomic hydrogen adsorption, thus providing a clearer understanding of the unique bonding and structural characteristics of the HB sheets.

## 1. Introduction

Owing to the unique properties and promising applications of two-dimensional materials, researches in this field have garnered significant interest and attention [1,2,3,4,5]. Since the successful discovery and study of various 2D materials, such as graphene (a single layer of carbon atoms [6,7,8]), numerous efforts have been made to produce and synthesize a diverse range of 2D materials with varying physical properties, including as insulators, semiconductors, and superconductors [2,3,4,5]. Combining these two-dimensional materials can substantially enhance the performance of electronic devices, create novel materials with intriguing new physical properties, and enable the exploration of quantum phenomena [6,7,8,9,10,11,12]. For instance, transistors, which are crucial components in electronic devices that control electrical signals, can be fabricated from 2D materials [8,9,10]. These nanoscale transistors benefit from the planar surfaces of 2D materials, which are essential for optimal performance.

As the neighboring element to carbon in the periodic table, boron, with one less electron and a lower atomic mass than carbon, can exist in different allotropes and structures, such as bulk boron, borophene, boron clusters, and even one-dimensional boron chains [13,14,15,16]. In comparison to the well-known two-dimensional carbon materials, including graphite and graphene, boron is capable of forming various 2D structures akin to those of carbon [1,13,14,15,16,17]. Consequently, 2D boron-based materials continue to stimulate increasing attraction and research. Additionally, metallic boride crystals, such as MgB_2_, exhibit stable planar 2D structures, providing evidence for the potential synthesis of boron-based 2D materials analogous to graphene [18,19]. Borophene, a promising candidate for ideal 2D boron-based materials, also forms a layered structure with a few hexagonal boron layers. It was first synthesized on Ag (111) in 2015 [20] and has been produced on other metal surfaces [21,22,23,24]. Scanning tunneling microscopy (STM) images and theoretical calculations have confirmed the layered, planar structure of these boron materials [15,20,21]. After synthesizing and confirming the structure of borophene, studies have also explored its interlayer stacking properties [25,26,27]. However, an unresolved issue is that borophene reportedly cannot remain stable without a metal substrate and is reactive with water and oxygen in the air [28,29].

To avoid the unstable characteristics of borophene, hydrogen boride (HB) sheet, which is also a boron-based 2D material, has attracted more interest for its stability even in an aqueous condition [30]. The ideal structure of HB sheets has been theoretically calculated using first-principles methods to determine their theoretical discharge capacity. Results indicate that the discharge capacity of HB sheets is exceptionally high compared to graphite anodes [31,32,33,34,35,36]. Additionally, the stability of HB sheets has been examined under aqueous conditions, demonstrating that they remain stable [36]. Studies on the hydrogen storage capability of HB sheets are also advancing, with investigations into the release of gaseous H_2_ under photoirradiation [37]. Furthermore, HB sheets are considered as excellent substrates for the formation of highly dispersed Ni nanoclusters [38]. Building on previous work, reducing the byproduct of boric acid could be a valuable and meaningful research avenue, potentially enhancing the purity and quality of HB sheets [39].

To understand the properties of materials, X-ray photoelectron spectroscopy (XPS) is commonly employed and often combined with complementary techniques, such as Fourier Transform Infrared (FT–IR) spectroscopy and Transmission Electron Microscopy (TEM) imagery [37,38,39,40]. By analyzing the B 1s core-level spectra of boron materials, the typical peak positions and assignments for various boron species have been reported and studied, providing a reliable reference for boron XPS research [37,38,39,40,41]. XPS can also be used to track changes in chemical states following different reactions, including oxidation, reduction, and adsorption processes on material surfaces. These changes are reflected in shifts in peak positions and variations in peak area ratios in the XPS spectra. Alterations in component peaks can indicate possible reactions occurring during the process and provide insights into the species changes of HB sheets and bulk boron. The purpose of this research is to focus on the chemical bonding and chemical states of the boron atoms on the HB sheets’ surface, and to verify the possible reactions caused by the boron species’ bonding, by applying XPS.

## 2. Materials and Methods

Hydrogen boride sheets are prepared via an ion-exchange reaction. Specifically, 1 g of MgB_2_ black crystal powders (99%, Rare Metallic Co., Ltd., Tokyo, Japan) was stirred at 350 rpm with 45 mL of HCl-treated ion-exchange resin (Amberlite IR120B H HG, Organo Corp., Tokyo, Japan) for 3 days at room temperature (293 K). The solvent used in this process was 300 mL acetonitrile (99.5%, JIS special grade, FUJIFILM Wako Pure Chemical Corp, Osaka, Japan) in a Schlenk flask under an argon atmosphere. It is crucial to avoid exposing the reactants to air, as the process is sensitive to water, which can cause hydrolysis. After stirring for 3 days, the mixture was cooled to 273 K overnight to allow the deposition of unreacted MgB_2_, ion-exchange resin, and byproduct boric acid. The mixture was then filtered using a 0.2 μm membrane filter paper under a flowing argon atmosphere. The resulting HB sheets were obtained by drying the solvent in an oil bath at 343 K, with a cold trap to remove the evaporated acetonitrile.

FT–IR spectroscopy was measured in Ar atmosphere under room temperature with the scanning range of 400–4000 cm^−1^. The FT–IR results were recorded by the attenuated total reflectance (ATR) method using prism holders. TEM images were obtained in vacuum condition under acceleration electron-beam voltage at 200 kV.

Analysis and measurement of chemical states of the elements were performed by XPS at beamline (BL-13B) at the Photon Factory, KEK, at room temperature with photon energies of 285 and 630 eV; photon energy resolution (E/∆E) of BL-13B at the silt width of 30 μm was estimated as 10,000 at photon energy of 64.1 eV and 7200 at 867 eV [42]. Both bulk boron and HB sheets were prepared under an Ar atmosphere and baked under 373 K for 3 days to degas the remaining moisture on the surfaces. The XPS electron analyzer was equipped in the main UHV chamber (Gamma Data/Scienta, SES200) and the pass energy of the measurements was 75 eV. The reproducibility experiment was held at beamline (BL08U) at Nano Terasu.

Both bulk boron and HB sheets were exposed to the atomic hydrogen atmosphere, which can be obtained by cracking the molecular hydrogen gas by a heated tungsten filament [43], with cracking condition is under a pressure of 5.0 × 10^−6^ Torr and at room temperature. The heating filament current was set at 6.5 A for 10 min. For both HB sheets and bulk boron, the atomic hydrogen-adsorbed volume was set to 3000 Langmuir (L).

## 3. Results and Discussion

### 3.1. FT–IR and TEM

**Figure 1:**
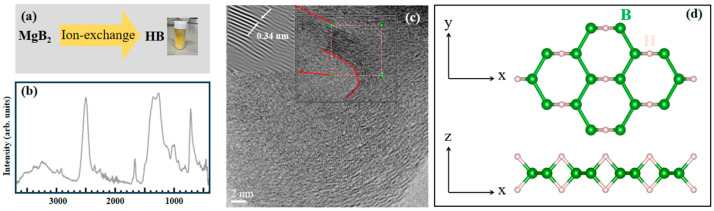
(**a**) Schematic synthesizing process of HB sheets by ion-exchange. (**b**) FT–IR spectrum of HB sheets. (**c**) TEM images of HB sheets; inset shows the region enclosed by the red square. A winding structure is traced by a red curve. (**d**) Structure model of HB sheets.

Figure 1a shows the HB sheets synthesized through an ion-exchange reaction involving metal borides and protons, with MgB_2_ crystals as the metal boride used in this study.

To examine the structure and study the properties of the HB sheets, FT–IR and TEM were employed, with the results shown in Figure 1b,c, respectively. Figure 1d displays the 3D structure model of the HB sheets [1,37,38,39,40,41,44]. Figure 1b presents the FT–IR spectrum of the HB sheets, which exhibits a prominent absorption peak at 2500 cm^−1^, attributed to a stretching mode at the terminal B-H bonds at sheet edges. Peaks at 1400, and 1650 cm^−1^ correspond to bridging vibration modes and the peak at 700 cm^−1^ is attributed to the angular oscillation at the B-H-B bond, while the peak at 1000 cm^−1^ is associated with a stretching mode of the B-H bond with angular oscillations. The spectrum also shows an O-H stretching peak above 3000 cm^−1^, indicating the presence of residual boric acid [37,38,39,40,41]. Figure 1c displays the TEM image, which confirms the sheet-like structure of the HB. By focusing on the enclosed area, the interlayer distance was measured to be approximately 0.34 nm, which was consistent with previous studies [38]. Additionally, the TEM image reveals a winding structure highlighted by the red curve, suggesting that the HB sheets are not as flat as the structure model. Both the FT–IR spectrum and TEM image confirm the successful synthesis of the HB sheets.

Analyzing the structure of the HB sheets from Figure 1b,c, they consist of layered structures, similar to those in an MgB_2_ crystal [37,38,39,40,41]. Observations of vibrational modes in the FT–IR spectrum further confirm that there are B-H-B and B-H bonds within the HB sheets. The B-H-B bond can be characterized as a three center/two electron (3c–2e) bond at either surface side of the HB sheet [37,38,39,40,41]. As shown in Figure 1c, the HB sheets are predominantly fragmented and exhibit a winding structure.

### 3.2. XPS

XPS is a powerful technique for studying and characterizing the chemical elements on the surface of samples. Additionally, XPS B 1s core-level spectra can reveal changes in charge states after chemical reactions. By calculating the atomic charges of B and H atoms in HB sheets using Löwdin population analysis, the result indicated that the B atoms in the pristine HB sheets were negatively charged, as their number was slightly higher than the total valency [30]. This conclusion can also be confirmed by the observation that, if B atoms were positively charged, negatively charged O atoms of water would be expected to be closer to B atoms, rather than positively charged H atoms [30]. Based on these results, we can predict that, in the XPS B 1s core-level spectra of HB sheets, the peaks for B species (B-H-B and B-H) should appear in a lower binding energy region compared to the B 1s core-level spectra of bulk boron, which typically shows B-B bonding.

**Figure 2:**
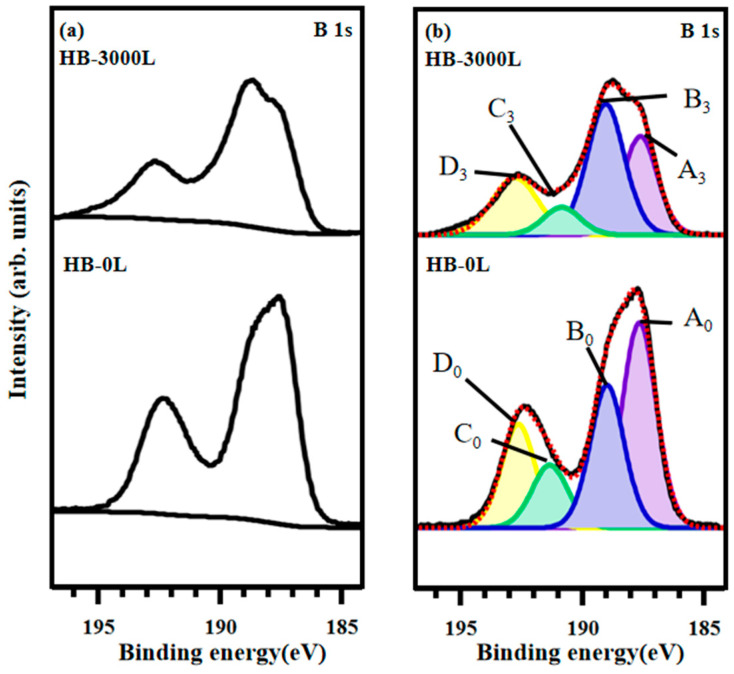
XPS B 1s core-level spectra for the HB sheets with photon energy of 285 eV. (**a**) Background subtraction of XPS B 1s spectra: 3000 L atomic hydrogen adsorbed on HB sheets (**above**) and initial HB sheets (**below**). (**b**) Curve fitting of the B 1s XPS spectra.

Figure 2a displays the XPS B 1s core-level spectra of the HB sheets, measured at 285 eV. Figure 2b shows the background-subtracted spectra for both the initial and 3000 Langmuir (L) atomic hydrogen-adsorbed HB sheets. The background subtraction was performed using the Shirley type function. In Figure 2b, the pristine data are presented as black lines, and the curve-fitted XPS spectra are shown as red dashed lines. Fitting of an XPS spectrum was made based on a function that combines the Gaussian (G) and Lorentzian (L) functions with software CASAXPS [45,46]. In line shape analysis, a model for fitting asymmetric peaks as a Lorentzian form convoluted with a Gaussian form is necessary, since most of the broadening in core-level spectra arises from the analyzer, X-ray source, and other final-state interactions, which are best represented by Gaussian distributions [47,48].

Both the initial and atomic hydrogen-adsorbed spectra are fitted with four component peaks, and the line shape of the fitting peaks is GL (30) for 30% Lorentzian and 70% Gaussian. These peaks are labeled A_0_–D_0_ for the initial state and A_3_–D_3_ for the 3000 L hydrogen-adsorbed state. The different letters indicate distinct species, while the subscripts denote the condition of atomic hydrogen adsorption, with 0 representing 0 L and 3 representing 3000 L. Peak positions, assignments, and area ratios are summarized in Table 1. Based on analysis of binding energies, taken at the measurement conditions, the four peaks can be attributed to boron species at B-H-B bonding site in HB (A_0.3_), at B-H bonding site in HB (B_0.3_), at B-OH bonding site in boric acid, B(OH)_3_ (D_0.3_), and at a site of an intermediate boron material between HB and B(OH)_3_ (C_0.3_) [37,38,39,40,41,49,50,51,52].

Further analysis of the results shown in Figure 2 reveals that both the initial and atomic hydrogen-adsorbed HB sheets exhibit a decrease in the A peak. The reduction in an area ratio of the A peak indicated a decrease in the B-H-B bridging network structure on the surface of the HB sheets. Concurrently, the increase in the B peak, assigned to the B-H bond, suggests that the breaking of the B-H-B bonds results in the formation of the B-H bonds by the hydrogeneration. The C and D peaks are located at a binding energy region of positively charged boron, considered as B-OH bonds in oxidized boron, such as boric acid. Positions of the D peak can originate from B(OH)_3_, while those of the C peak may be from B(OH) or B(OH)_2_ sites in the HB sheet.

**Figure 3:**
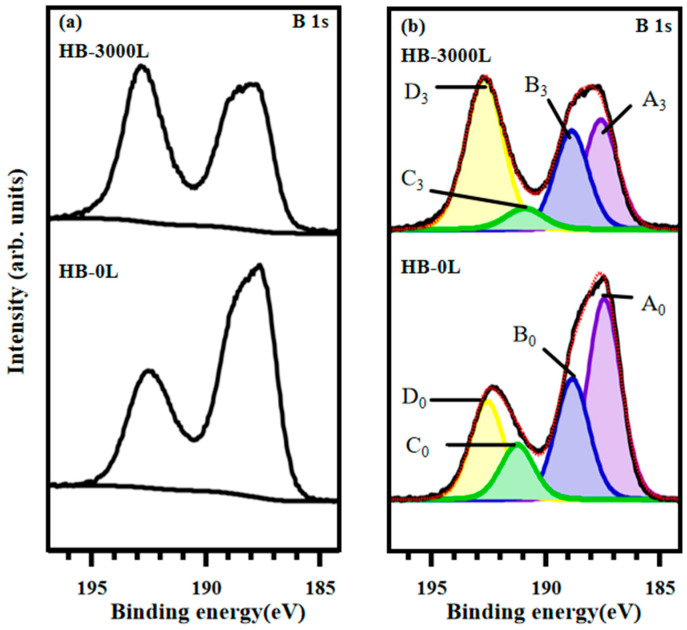
XPS B 1s core-level spectra for the HB sheets with photon energy of 630 eV. (**a**) Background subtraction of XPS B 1s spectra: 3000 L atomic hydrogen adsorbed on HB sheets (**above**) and initial HB sheets (**below**). (**b**) Curve fitting of the B 1s XPS spectra.

To extensively explore changes in chemical state in the HB sheet, we also investigated the XPS B 1s core-level spectra with a photon energy of 630 eV. The measurement condition provides the bulk-sensitive information in XPS, as compared to that of the photon energy at 285 eV. The XPS B 1s core-level spectra of HB sheets, taken at a photon energy of 630 eV, are presented in Figure 3a,b. Figure 3a shows the background-subtracted spectra for both the pristine and 3000 L atomic hydrogen-adsorbed HB sheets, with background subtraction performed using the Shirley type. Figure 3b displays the experimental data as black lines and the curve-fitted spectra as red dashed lines. Both sets of curve-fitted spectra are also decomposed into four component peaks.

The four component peaks are labeled as A_0_–D_0_ for the pristine state and A_3_–D_3_ for the 3000 L atomic hydrogen-adsorbed state. Comparison with Figure 2b shows that the peak binding energy positions are consistent, indicating that the peak fitting is accurate, and the identified species are consistent across different photon energies. Assignments and area ratios of the four species of peaks are summarized in Table 2.

To further discuss the results shown in Figure 3, a decrease in the A peak is also observed, which indicates a reduction in the B-H-B bridging network structure on the surface of the HB sheets. Simultaneously, the increase in the B-H peak area ratio confirms that the breaking of B-H-B bonds leads to the formation of additional B-H bonds after hydrogenation. From Table 2, we observe a decrease in the A peak and increases in both the B and D peaks. Compared to the spectra obtained with 285 eV photon energy, the increase in the D peak is pronounced at 630 eV. This enhanced increase is attributed to the presence of oxygen in the bulk structure of the HB sheets, which reacts with the broken B-H-B bridging bonds, resulting in a greater formation of positively charged boron and boric acid compared to the 285 eV spectra. The results confirm that atomic hydrogen adsorption breaks the B-H-B bonds, forming more B-H bonds. Additionally, oxygen in the deeper bulk structure of the HB sheets reacts with the broken bridging B-H-B bonds, leading to a notable increase in boric acid. Moreover, the slight increase in B-H peak area ratio indicates the surface sensitive characteristics of the B-H bonding. For our experiment, since the incoming reactant is atomic hydrogen, instead of molecular hydrogen, the reaction process can be described as an Eley–Ridealtype. The Eley–Rideal mechanism is a direct reaction between the incoming molecule and the reactant at a surface, followed by the product desorption [53,54,55]. In the current case, atomic hydrogen twice reacts with the bridging B-H-B bonds, resulting in new B-H bonds.

**Figure 4:**
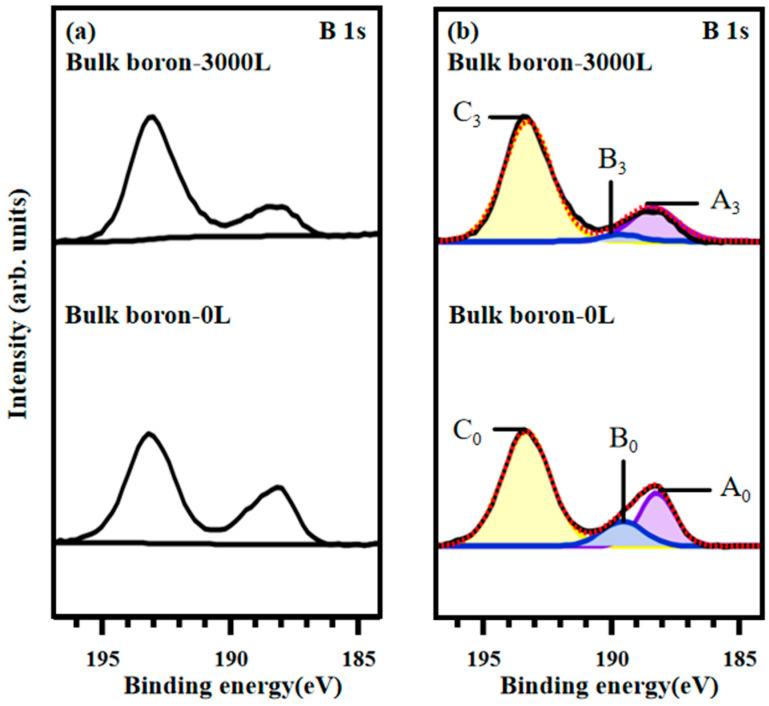
XPS B 1s core-level spectra for bulk boron with photon energy of 285 eV. (**a**) Background subtraction of XPS B 1s spectra: 3000 L atomic hydrogen adsorbed on HB sheets (**above**) and initial HB sheets (**below**). (**b**) Curve fitting of the B 1s XPS spectra.

To verify that B-H bonds are formed by breaking of the B-H-B bonds rather than by direct interaction of boron with atomic hydrogen, we applied hydrogeneration to the bulk boron and made XPS measurements to compare the results with the HB sheets. The XPS B1s core-level spectra of bulk boron at 285 eV are shown in Figure 4a,b. The curve-fitted spectra are decomposed into three component peaks, labeled A_0_–C_0_ for initial state and A_3_–C_3_ for 3000 L hydrogen-adsorbed state. The peak positions, the assignments, and the area ratios are given in Table 1.

In the spectra with 285 eV photon energy shown in Figure 4b, it is evident that the bulk boron sample does not react with atomic hydrogen, as the peak positions for each component (A to C) remain unchanged. This lack of change in peak positions suggests that the chemical state of boron on the surface of bulk boron does not alter after exposure to atomic hydrogen. Additionally, the area ratios of each peak show minimal variation following atomic hydrogen exposure. Specifically, the decrease in the B-O bonding peak area ratio and the increase in the boric acid peak area ratio suggest that some transformation reactions between B-O and boric acid might occur during atomic hydrogen treatment. These results indicate that the formation of new B-H bonds in the HB sheets is not a direct result of boron interacting with atomic hydrogen. Instead, it arises from the breaking of B-H-B bridging bonds, which are not present in the structure of bulk boron.

**Figure 5:**
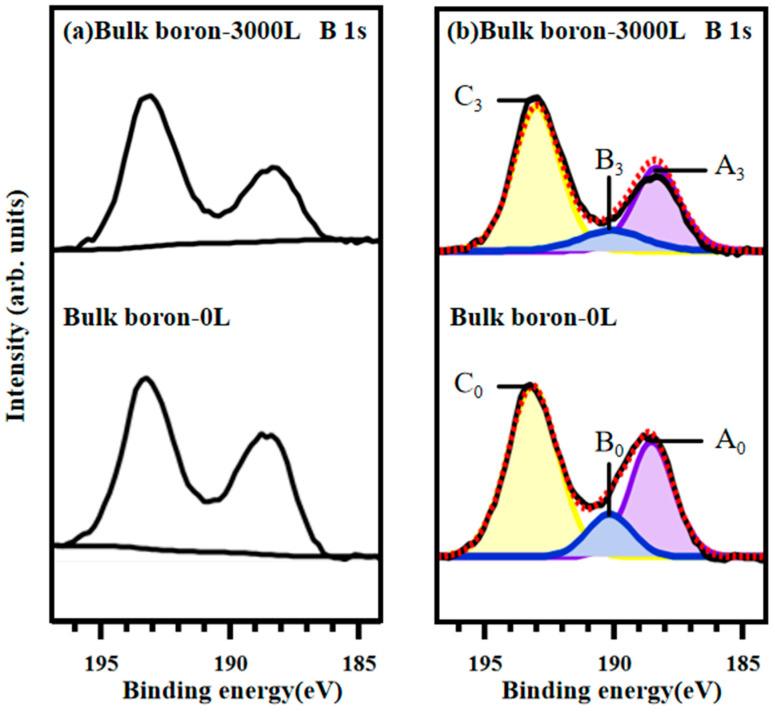
XPS B 1s core-level spectra for bulk boron with photon energy of 630 eV. (**a**) Background subtraction of XPS B 1s spectra: 3000 L atomic hydrogen adsorbed on HB sheets (**above**) and initial HB sheets (**below**). (**b**) Curve fitting of the B 1s XPS spectra.

The XPS B 1s core-level spectra of bulk boron at 630 eV are also presented in Figure 5a,b. Figure 5a shows the background-subtracted spectra for both the initial and 3000 L atomic hydrogen-adsorbed bulk boron. In Figure 5b, the raw data are depicted as black lines, and the curve-fitted spectra are shown as red dashed lines. Both sets of curve-fitted spectra are decomposed into three component peaks. Analysis of the binding energies and measurement conditions reveals that the three species peaks are attributed to B-B bonds, B-O bonds, and boric acid. Compared to Figure 4b, the background interference peak is absent at 630 eV, while the positions of the other three component peaks are consistent with those observed at 285 eV. The peak positions, assignments, and area ratios are summarized in Table 2.

The spectra obtained with 630 eV photon energy, shown in Figure 5b, also indicate that the bulk boron sample does not react with atomic hydrogen, as the peak positions for each component (A to C) remain unchanged. This lack of change in peak positions agrees with the results under 285 eV, which suggests that the chemical state of boron on the surface of the bulk boron does not alter after atomic hydrogen adsorption. Moreover, the area ratios of each peak show minimal variation following atomic hydrogen treatment. These results further confirm that the formation of new B-H bonds is not due to a direct reaction between boron and atomic hydrogen. Instead, it arises from the breaking of B-H-B bridging bonds, which are absent in the bulk boron structure.

The present method of atomic hydrogen adsorption paves a way to control a ratio between numbers of the B-H-B and B-H bonds in a HB sheet. It has been reported that the optical properties of the HB sheets depend on the B-H-B/B-H bond ratio [56]. An increase in the ratio enhances the luminescence intensity, while a decrease enhances the photoinduced H_2_ release rate under UV irradiation. We have found that the hydrogenation reduces the B-H-B/B-H bond ratio and thus the method is suitable to tune the HB sheets for the optimized H_2_ generation for usage. The present research has given not only academic insights into understanding the nature of the hydrogen surface chemistry at the atomic sheet but has also provided technological seeds for hydrogen production technology.

## 4. Conclusions

Hydrogen boride sheets were successfully synthesized via an ion-exchange reaction. Their structure and chemical bonding were examined using FT–IR and TEM. XPS B 1s core-level spectra of the HB sheets measurements were conducted at photon energies of 285 eV and 630 eV. For the surface sensitive measurement with 285 eV, the B 1s XPS spectra of the HB sheets were fitted into four component peaks, corresponding to B-H-B bonds in HB, B-H bonds in HB, B-OH bonds at the intermediate, and B-OH bonds in B(OH)_3_, respectively. After atomic hydrogeneration, the area ratio of the B-H-B bonding peak decreased, and the B-H bond peak increased. This result indicated that atomic hydrogen treatment led to the cleavage of the pristine B-H-B bonds and to the formation of the B-H bonds. For bulk sensitive measurement with a photon energy of 630 eV, decrease in a number of the B-H-B bond was also observed, but a number of the B-H bond increased slightly. This indicated rather that the B-H bonding formation was held at a surface. The XPS results also indicated that the breaking of B-H-B bonds led to an increase in the positively charged boron species, which suggested that the cleavage of B-H-B bonds has allowed its breakdown by the oxygen impurities in the internal bulk. In the case of bulk boron, XPS measurements revealed that the neutral boron atoms hardly reacted with the atomic hydrogen. The present study provides valuable insights into the chemical sensitivity of the B-H-B bond in HB sheets and paves a way to explore industrial reactions.

## Figures and Tables

**Table 1:** XPS fitting results for the HB sheets and bulk boron for initial and atomic hydrogen-adsorbed conditions with photon energy of 285 eV.

Samples	Component	Peak Position (eV)	Peak Assignment	Area Rate%	FWHM (eV)	Area Relative Error %
HB-0 L	A_0_	187.67	B-H-B bonding sitein HB	40.78	1.70	0.08
	B_0_	189.02	B-H bonding sitein HB	25.05	1.70	0.06
	C_0_	191.47	B-OH bonding sitein intermediate	7.69	1.70	0.08
	D_0_	192.80	B-OH bonding sitein B(OH)_3_	26.48	1.70	0.12
HB-3000 L	A_3_	187.60	B-H-B bonding sitein HB	24.81	1.84	0.09
	B_3_	189.10	B-H bonding sitein HB	43.63	1.84	0.17
	C_3_	191.00	B-OH bonding sitein intermediate	7.49	1.86	0.17
	D_3_	192.95	B-OH bonding sitein B(OH)_3_	24.07	1.84	0.17
Bulk boron-0 L	A_0_	188.11	B-B bonding sitein boron	21.82	1.87	0.16
	B_0_	189.41	B-O bonding sitein oxidized boron	12.47	1.89	0.17
	C_0_	193.11	B-OH bonding sitein oxidized boron	65.71	1.92	0.13
Bulk boron-3000 L	A_3_	188.00	B-B bonding sitein boron	21.01	2.14	0.17
	B_3_	189.85	B-O bonding sitein oxidized boron	3.36	2.12	0.17
	C_3_	193.52	B-OH bonding sitein oxidized boron	74.63	2.14	0.18

**Table 2:** XPS fitting results for HB sheets and bulk boron for initial and atomic hydrogen-adsorbed conditions with photon energy of 630 eV.

Samples	Component	Peak Position (eV)	Peak Assignment	Area Rate%	FWHM (eV)	Area Relative Error %
HB-0 L	A_0_	187.50	B-H-B bonding site in HB	43.02	1.68	0.14
	B_0_	188.87	B-H bonding site in HB	25.05	1.68	0.09
	C_0_	191.21	B-OH bonding site in intermediate	7.69	1.68	0.08
	D_0_	192.52	B-OH bonding site in B(OH)_3_	26.48	1.68	0.11
HB-3000 L	A_3_	187.58	B-H-B bonding site in HB	26.51	1.92	0.11
	B_3_	188.74	B-H bonding site in HB	25.98	1.92	0.10
	C_3_	190.91	B-OH bonding site in intermediate	5.83	1.94	0.10
	D_3_	192.61	B-OH bonding site in B(OH)_3_	41.67	1.92	0.13
Bulk boron-0 L	A_0_	188.26	B-B bonding site in boron	32.77	2.27	0.15
	B_0_	189.90	B-O bonding site in oxidized boron	12.14	2.27	0.17
	C_0_	192.91	B-OH bonding site in oxidized boron	55.09	2.27	0.15
Bulk boron-3000 L	A_3_	188.00	B-B bonding site in boron	32.57	2.16	0.30
	B_3_	189.85	B-O bonding site in oxidized boron	10.75	2.16	0.28
	C_3_	193.52	B-OH bonding site in oxidized boron	56.68	2.08	0.21

## Data Availability

Data are contained in the article.
